# Stimulation of IL-1β and IL-6 through NF-κB and sonic hedgehog-dependent pathways in mouse astrocytes by excretory/secretory products of fifth-stage larval *Angiostrongylus cantonensis*

**DOI:** 10.1186/s13071-017-2385-0

**Published:** 2017-09-26

**Authors:** Kuang-Yao Chen, Lian-Chen Wang

**Affiliations:** 1 Department of Parasitology, College of Medicine, Chang Gung University, Taoyuan, 333 Taiwan; 2 Graduate Institute of Biomedical Sciences, College of Medicine, Chang Gung University, Taoyuan, 333 Taiwan; 3 Molecular Infectious Disease Research Centre, Chang Gung Memorial Hospital, Taoyuan, Taiwan

**Keywords:** *Angiostrongylus cantonensis*, Excretory/secretory products, Astrocytes, Sonic hedgehog signaling pathway, NF-κB, Cytokine

## Abstract

**Background:**

*Angiostrongylus cantonensis* is an important causative agent of eosinophilic meningitis and eosinophilic meningoencephalitis in humans. Previous studies have shown that the Sonic hedgehog (Shh) signaling pathway may reduce cell apoptosis by inhibiting oxidative stress in *A. cantonensis* infection. In this study, we investigated the relationship between cytokine secretion and Shh pathway activation after treatment with excretory/secretory products (ESP) of fifth-stage larval *A. cantonensis* (L5).

**Results:**

The results showed that IL-1β and IL-6 levels in mouse astrocytes were increased. Moreover, ESP stimulated the protein expression of Shh pathway molecules, including Shh, Ptch, Smo and Gli-1, and induced IL-1β and IL-6 secretion. The transcription factor nuclear factor-κB (NF-κB) plays an important role in inflammation, and it regulates the expression of proinflammatory genes, including cytokines and chemokines, such as IL-1β and TNF-α. After ESP treatment, NF-κB induced IL-1β and IL-6 secretion in astrocytes by activating the Shh signaling pathway.

**Conclusions:**

Overall, the data presented in this study showed that ESP of fifth-stage larval *A. cantonensis* stimulates astrocyte activation and cytokine generation through NF-κB and the Shh signaling pathway.

**Electronic supplementary material:**

The online version of this article (10.1186/s13071-017-2385-0) contains supplementary material, which is available to authorized users.

## Background

Astrocytes are the most abundant cells in the human and murine central nervous system (CNS). These cells can protect neurons and induce inflammatory responses by releasing anti-apoptotic proteins or cytokines in response to pathogen infection [1, 2]. During infection, the activated astrocytes generate IL-1β and IL-6 through the p38/IκB- or TNFα/NF-κB-mediated pathway [3–6] and delay neuronal death in pathological situations with H_2_O_2_ generation [7]. Astrocytes also form the blood-brain barrier with endothelial cells to regulate molecular transportation [8]. This barrier protects the CNS by separating the blood and brain cells, and it only allows specific small molecules, such as O_2_, CO_2_, and glucose, to cross into brain tissue [9]. Several studies have shown that approximately 100% of large-molecule drugs and more than 98% of small-molecule drugs cannot reach the brain through the blood-brain barrier. Viruses are also required to cross this barrier to infect brain cells *via* induction of cell death in astrocytes [10].

The Hedgehog (Hh) signaling pathway and secreted proteins play important roles in animal development. This pathway regulates morphogenesis of a variety of tissues and organs [11]. Hh has three homologs, including Sonic hedgehog (Shh), Desert hedgehog (Dhh) and Indian hedgehog (Ihh) [12]. Shh signaling is mediated *via* a series of inhibitory steps, and it can trigger a common signaling pathway. In the absence of Shh, transmembrane Patched (Ptc) receptors block the function of another transmembrane protein, Smoothened (Smo). In contrast, following Shh interaction with Ptc, Smo can be activated *via* inhibition of Ptc. These changes initiate a signaling cascade that activates the Glioma-associated oncogene (Gli) family of transcription factors (Gli1-Gli3) [13, 14]. Several studies have shown that cytokine expression in response to infections is stimulated by Shh signaling. In *Helicobacter pylori* infection, the Shh pathway can positively regulate the expression of Interleukin-1β (IL-1β), IL-10, IL-12, IFNγ and MIP-2 in mouse stomach tissues [15]. IL-1β is a pro-inflammatory cytokine that plays an important role in brain inflammation and promotes the production of other cytokines, such as TNF-α and IL-6, in microglia and astrocytes [16, 17]. IL-1β and IL-6 play an important role in CNS immune responses. The expression levels of IL-1β and IL-6 in brains are increased on brain injury, parasites infection and autoimmune encephalomyelitis [18–20].

*Angiostrongylus cantonensis*, the rat lungworm, was found in the hearts and pulmonary arteries of *Rattus rattus* and *Rattus norvegicus* in Guangzhou (Canton), China, by Chen in 1935 [21, 22]. This parasite is an important causative agent of human cerebral angiostrongyliasis (eosinophilic meningitis and eosinophilic meningoencephalitis), particularly in the Pacific islands and Southeast Asia [23–28]. By 2010, more than 3000 cases had been reported in approximately 30 countries worldwide [29, 30]. In *A. cantonensis* infection, the fifth-stage larvae (L5) induce a wide range of immune responses, including eosinophil recruitment and cytokine release (IL-1β, IL-4, IL-5, IL-6, IL-10, IL-13 and TNF-α) in the CNS of humans [31, 32].

In our previous studies, *A. cantonensis* infection in mice increased reactive oxygen species (ROS) and antioxidants in the astrocytes, and activation of the Shh signaling pathway inhibited cell death through the GRP78/Bcl-2-dependent pathway [33]. Moreover, excretory/secretory products (ESP) from *A. cantonensis* L5 induced oxidative stress and cell apoptosis in astrocytes, but Shh pathway activation could reduce cell injury [34]. In the present study, we reported that ESP increases IL-1β and IL-6 levels in mouse astrocytes in a time-dependent manner, and the NF-κB/Shh pathway plays an important role in cytokine secretion.

## Methods

### Parasite and experimental infection

*Angiostrongylus cantonensis* (Taipei strain) was maintained in our laboratory through cycling in *Biomphalaria glabrata* snails and Sprague-Dawley (SD) rats [33]. On day 21 post-infection, the third-stage larvae (L3) were isolated from the infected snails by digestion with 0.6% (*w*/*v*) pepsin-HCl (pH 2–3) for 1 h. Each BALB/c mouse was inoculated with 25 L3 *via* stomach intubation. In this study, SD rats and BALB/c mice were purchased from the National Laboratory Animal Center, Taipei. All procedures involving animals and their care were reviewed and approved by the Chang Gung University Institutional Animal Care and Use Committee.

### Preparation of *A. cantonensis* ESP

Live L5 of *A. cantonensis* were isolated from the brain tissues of rats by anesthetizing with 30 μl Zoletil 50 (Virbac) after 21 days post infection. After the worms were washed with saline, phosphate-buffered saline, distilled water and RPMI (Sigma-Aldrich, St. Louis, USA), they were incubated in RPMI without foetal bovine serum (FBS) for 24, 48 and 72 h. The ESP of L5 were collected and concentrated by Amicon Ultra-15 10 K centrifugal filter devices (Merck Millipore, Darmstadt, Germany) from the culture medium. The ESP concentration in the medium was determined using a Bio-Rad Protein Assay Kit (Bio-Rad, Hercules, CA, USA), according to the manufacturer’s instructions. The ESP were used to treat the astrocytes, and cellular changes were observed [34].

### Astrocyte culture

A mouse brain astrocytic cell line (CRL2535) from ATCC was used in this study [31]. Cells were cultured in Dulbecco’s modified Eagle’s medium/F-12 (DMEM/F-12) (Corning, New York, USA) with 10% fetal bovine serum (FBS) (Gibco, Waltham, USA), penicillin and streptomycin in poly-L-lysine-coated culture flasks at 37 °C in 5% CO_2_. Cells were plated onto 10 cm culture dish, incubated in serum-free DMEM/F-12 for 24 h, pretreated with the the recombinant Shh (r-Shh) (R&D System, Minneapolis, USA), Shh agonist (SAG) (Enzo, New York, USA), Shh pathway inhibitor (Cyclopamine) (Sigma-Aldrich) and NF-Kb inhibitor (JSH-23) (Sigma-Aldrich) for 1 h and then treated with L5-ESP.

### SDS-PAGE electrophoresis and western blotting

The proteins of astrocytes were separated by 12% SDS-PAGE. The separated proteins were transferred to nitrocellulose membrane and incubated with antibodies against Shh (Sigma-Aldrich), Ptch (Sigma-Aldrich), Smo (Sigma-Aldrich), Gli-1 (Sigma-Aldrich), IL-1β (RayBiotech, Norcross, USA), IL-6 (RayBiotech), NF-κB (Sigma-Aldrich) and β-actin (Sigma-Aldrich). The membrane was washed with TBS/T three times and then incubated with a 1:10,000 dilution of anti-rabbit or mouse horseradish peroxidase antibody (Sigma-Aldrich). The reactive bands were detected by ECL reagents (EMD Millipore, Billerica, USA) and captured by a UVP BioSpectrum 600 Imaging System (Analytik Jena US, Upland, USA). ImageJ software analysis was used to detect the image densitometry of target proteins.

### ELISA

The cultured supernatants were collected from astrocytes treated with ESP at 2 h intervals, for up to 8 h. These samples were used to detect the concentration of IL-1β or IL-6 by a mouse-specific ELISA kit (RayBiotech).

### Quantitative real-time PCR

Total RNA was extracted from the astrocytes by using GENEzol TriRNA Pure Kit (Geneaid, Taipei, Taiwan). First -strand cDNA was synthesized using SuperScript III reverse transcriptase (Invitrogen, Carlsbad, USA) with oligo d(T) primer, according to the manufacturer’s instructions. Quantitative real-time PCR was performed using the qPCR Master Mix (KAPA, Wilmington, USA) on the ABI 700 qPCR System (Applied Biosystems, Foster City, USA). A β-actin internal control was used. The expression level was detected with specific primers, targeting the Shh (forward: 5′-GGC AGA TAT GAA GGG AAG AT-3′; reverse: 5′-ACT GCT CGA CCC TCA TAG TG-3′), Ptch (forward: 5′-GAA GGC GCT AAT GTT CTG AC-3′; reverse: 5′-TAC CTA GGA GGT ATG CTG TC-3′), Gli-1 (forward: 5′-TGC CAG ATA TGC TTC AGC CA-3′; reverse: 5′-TGT GGC GAA TAG ACA GAG GT-3′) and β-actin cDNAs (forward: 5′-CCT GTA TGC CTC TGG TCG TA-3′; reverse: 5′-CCA TCT CCT GCT CGA AGT CT-3′).

### Transfection of siRNA

The astrocytes were seeded into 6 well plates and grown to 70–80% confluence for transfection. X-tremeGene siRNA transfection reagent (Roche Molecular Systems Inc., Pleasanton, USA) was mixed with 40 pmoles Shh siRNA (UCUGAAACGCAGGACAAGG and CCUUGUCCUGCGUUUCAGA) (Sigma-Aldrich) within 5 min. Cells were incubated with the reagent containing siRNA for 8 h and then replaced the fetal bovine serum containing medium without siRNA. After 48 h, cells were collected to detect protein expression.

### Immunofluorescence staining

The frozen mouse brain tissue sections were fixed and permeabilized with 2% (*w*/*v*) paraformaldehyde (PFA) and 0.5% (*v*/v) Triton X-100 in PBS before incubation. Sections were washed in PBS/T (pH 7.4) and blocked for 30 min in PBS containing 2% BSA. Then, brain sections were incubated with primary antibody, including chicken anti-Glial fibrillary acidic protein(GFAP) (1:500, Abcam, Cambridge, UK) and rabbit anti-NF-κB, (1:50, Sigma-Aldrich), for 24 h at 4 °C. Finally, the sections were incubated with the secondary antibodies (DyLight™ 488–594-conjugated IgG, Jackson ImmunoResearch Inc., Newmarket, UK, 1:1000) for 50 min at room temperature. DAPI was used to detect cell nuclei.

### Statistical analysis

Student’s t-tests were used to compare the OD values and expression levels using GraphPad Prism 5 software. Data were presented as means ± SD. Differences were considered statistically significant through the *P*-value (*P < 0.05, **P < 0.01, ***P < 0.001).

## Results

### ESP induces IL-1β and IL-6 levels in mouse astrocytes

**Fig. 1:**
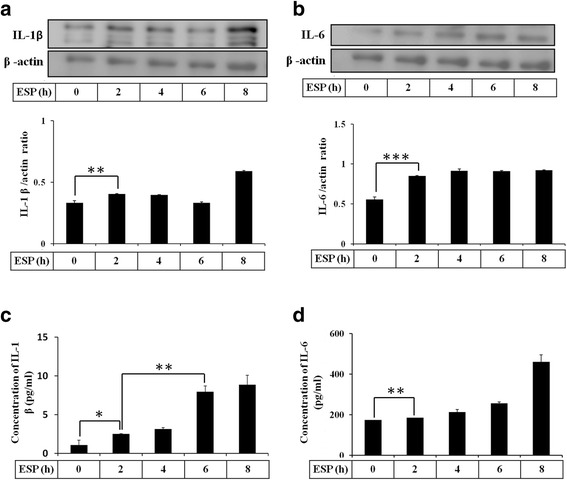
ESP induce IL-1β and IL-6. Astrocytes were treated with 500 μg/ml ESP at different time points. The protein levels of IL-1β (**a**) and IL-6 (**b**) were detected by Western blot analysis. The concentrations of IL-1β (**c**) and IL-6 (**d**) were determined in astrocyte culture medium by ELISA. Statistical significance was determined by Student’s *t*-test: *P < 0.05, **P < 0.01, ***P < 0.001 (*n* = 3)

To determine the role of *A. cantonensis* L5 ESP in cytokine expression, we treated astrocytes with 500 μg/ml ESP for 0–8 h. Western blot analysis showed that the protein levels of IL-1β and IL-6 increased in a time-dependent manner in astrocytes, and significant increases (IL-1β: *t*_(4)_ = 5.368, *P* < 0.01; IL-6: *t*_(4)_ = 147.8, *P* < 0.001) were detected at 2 h (Fig. 1a, b). Moreover, to assess whether the ESP induced cytokine secretion in astrocytes, we examined the protein concentrations of IL-1β and IL-6 in the culture medium by ELISA. The concentration of IL-1β in culture medium was increased (*t*_(4)_ = 3.978, *P* < 0.05) at 2 h and significantly increased at 6 h (*t*_(4)_ = 8.725, *P* < 0.01), and IL-6 was significantly increased (*t*_(4)_ = 4.893, *P* < 0.01) at 2 h (Fig. 1c, d). These results demonstrated that *A. cantonensis* L5 ESP induced IL-1β and IL-6 expression in astrocytes.

### ESP activates the Shh signaling pathway in mouse astrocytes

**Fig. 2:**
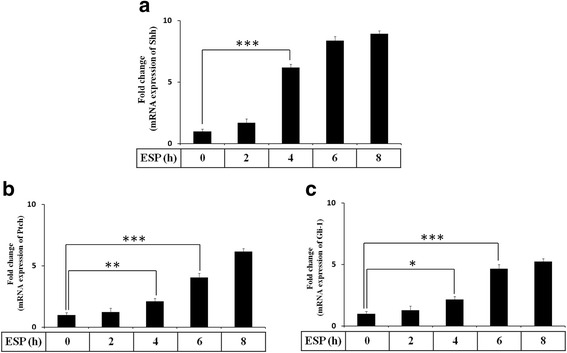
ESP stimulate the gene expression of Shh signaling pathway. Astrocytes were treated with 500 μg/ml ESP for the indicated time points. The mRNA levels of Shh, Ptch, and Gli-1 were determined in astrocytes by Quantitative real-time PCR. Statistical significance was determined by Student’s *t*-test: *P < 0.05, **P < 0.01, ***P < 0.001 (*n* = 3)

**Fig. 3:**
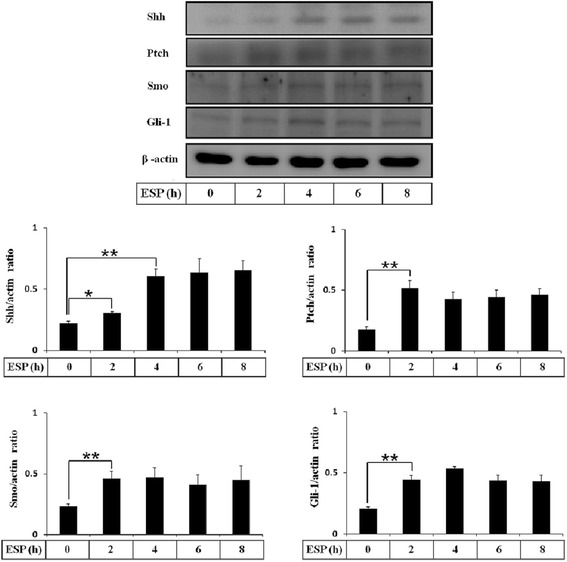
ESP induce Shh signaling pathway activation. Astrocytes were treated with 500 μg/ml ESP for the indicated time points. The protein levels of Shh, Ptch, Smo, and Gli-1 were determined in astrocytes by Western blots. Statistical significance was determined by Student’s *t-*test: *P < 0.05, **P < 0.01 (*n* = 3)

First, we wanted to determine whether Shh pathway was activated in standard medium treatment. These results showed that the protein levels of Shh pathway related molecules (Shh, Ptch, Smo, and Gli-1) were not significantly changed for 0–8 h in astrocytes (Additional file 1: Figure S1). Therefore, cell standard medium cannot induce the Shh pathway activation in astrocytes. In our previous studies, *A. cantonensis* L5 and soluble antigen induced astrocyte activation and Shh protein expression [33]. To determine whether the Shh signaling pathway is activated in astrocytes following treatment with *A. cantonensis* ESP, we assessed the mRNA expression level of Shh signaling cascade (Shh, Ptch, and Gli-1) members by quantitative real-time PCR (Fig. 2). The results showed that Shh was significantly elevated for 4 h (*t*_(4)_ = 16.68, *P* < 0.001), and Ptch and Gli-1 were significantly elevated for 6 h after ESP treatment (*t*_(4)_ = 13.24, *P* < 0.001). Moreover, the protein expression of Shh was significantly elevated for 4 h, and Ptch, Smo, and Gli-1 were significantly elevated for 2 h after ESP treatment (Shh: *t*_(4)_ = 6.187, *P* < 0.01; Ptch: *t*_(4)_ = 8.711, *P* < 0.01; Smo: *t*_(4)_ = 5.254, *P* < 0.01; Gli-1: *t*_(4)_ = 4.625, *P* < 0.01) (Fig. 3).

### Shh signaling pathway activation in mouse astrocytes increases IL-1β and IL-6 after ESP treatment

**Fig. 4:**
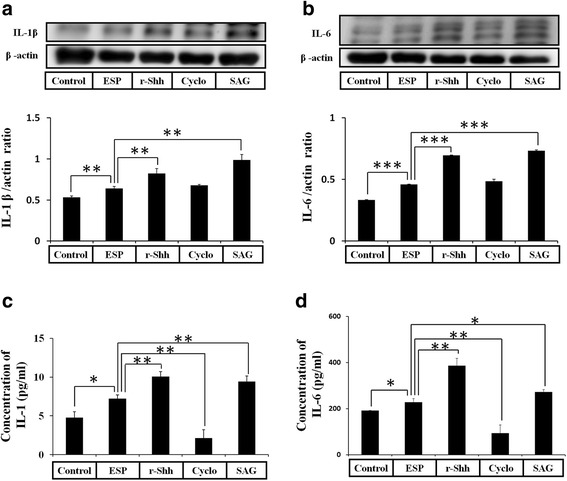
ESP induce the expressions of IL-1β and IL-6 *via* the Shh pathway. Western blot analysis of IL-1β (**a**) and IL-6 (**b**) in astrocytes co-cultured with ESP alone or pretreated with a recombinant Sonic hedgehog peptide from mouse (Shh) (3 μg), cyclopamine (20 μM) or SAG for 2 h and then with 500 μg/ml ESP for 4 h. β-actin is shown as a control. Data are expressed as the mean ± SD from three independent experiments (**P < 0.01). (**c**, **d)** Changes in the concentrations of IL-1β and IL-6 protein in the culture medium of astrocytes detected by the ELISA. Data are expressed as the mean ± SD from three independent experiments (*P < 0.05, **P < 0.01, ***P < 0.001)

**Fig. 5:**
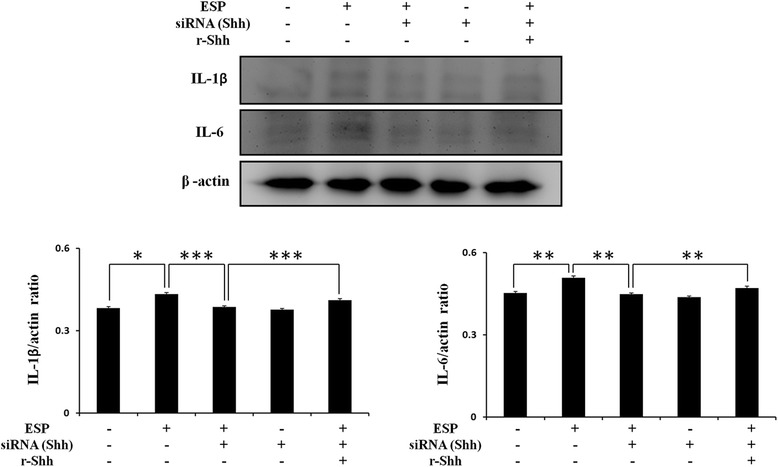
The expressions of IL-1β and IL-6 were increased through the Shh in ESP treatment. Western blot analysis of IL-1β and IL-6 in astrocytes co-cultured with ESP alone or pretreated with Shh siRNA for 48 h and then with a recombinant Sonic hedgehog peptide from mouse (Shh) (3 μg) for 2 h and 500 μg/ml ESP for 4 h. Data are expressed as the mean ± SD from three independent experiments (*P < 0.05, **P < 0.01, ***P < 0.001)

The Shh signaling pathway has been shown to regulate the immune response and cytokine release in human macrophages [35]. Therefore, we wanted to determine whether Shh signaling could stimulate cytokine production in ESP-treated astrocytes. In *A. cantonensis* ESP treatment experiments, the relationship between Shh signaling and cytokine generation in astrocytes was examined by Western blotting and ELISA following pretreatment with the recombinant Shh (r-Shh), Shh agonist (SAG), and Shh pathway inhibitor (Cyclopamine). These data showed that r-Shh and SAG stimulated IL-1β and IL-6 generation (IL-1β(r-Shh treatment): *t*_(4)_ = 4.884, *P* < 0.01; IL-1β(SAG treatment): *t*_(4)_ = 8.56, *P* < 0.01; IL-6(r-Shh treatment): *t*_(4)_ = 123.3, *P* < 0.001; IL-6(SAG treatment): *t*_(4)_ = 66.2, *P* < 0.001) (Fig. 4a, b) and secretion (IL-1β(r-Shh treatment): *t*_(4)_ = 5.948, *P* < 0.01; IL-1β(SAG treatment): *t*_(4)_ = 5.058, *P* < 0.01; IL-6(r-Shh treatment): *t*_(4)_ = 7.679, *P* < 0.01; IL-6(SAG treatment): *t*_(4)_ = 3.975, *P* < 0.01) (Fig. 4c, d) by activating the Shh pathway in ESP-treated astrocytes. Conversely, IL-1β and IL-6 expression were significantly decreased following inactivation of the Shh pathway by cyclopamine (IL-1β: *t*_(4)_ = 7.447, *P* < 0.01; IL-6: *t*_(4)_ = 5.912, *P* < 0.01). Alternatively, the results showed that the expressions of IL-1β and IL-6 were significantly decreased in Shh siRNA treatment (IL-1β: *t*_(4)_ = 11.71, *P* < 0.001; IL-6: *t*_(4)_ = 5.88, *P* < 0.01), and the expression levels could be restored after r-Shh treatment (IL-1β: *t*_(4)_ = 11.83, *P* < 0.001; IL-6: *t*_(4)_ = 7.435, *P* < 0.01) (Fig. 5). These results confirmed that the ESP elevated IL-1β and IL-6 through the Shh signaling pathway in astrocytes.

### *Angiostrongylus cantonensis* induces NF-κB expression in astrocytes

**Fig. 6:**
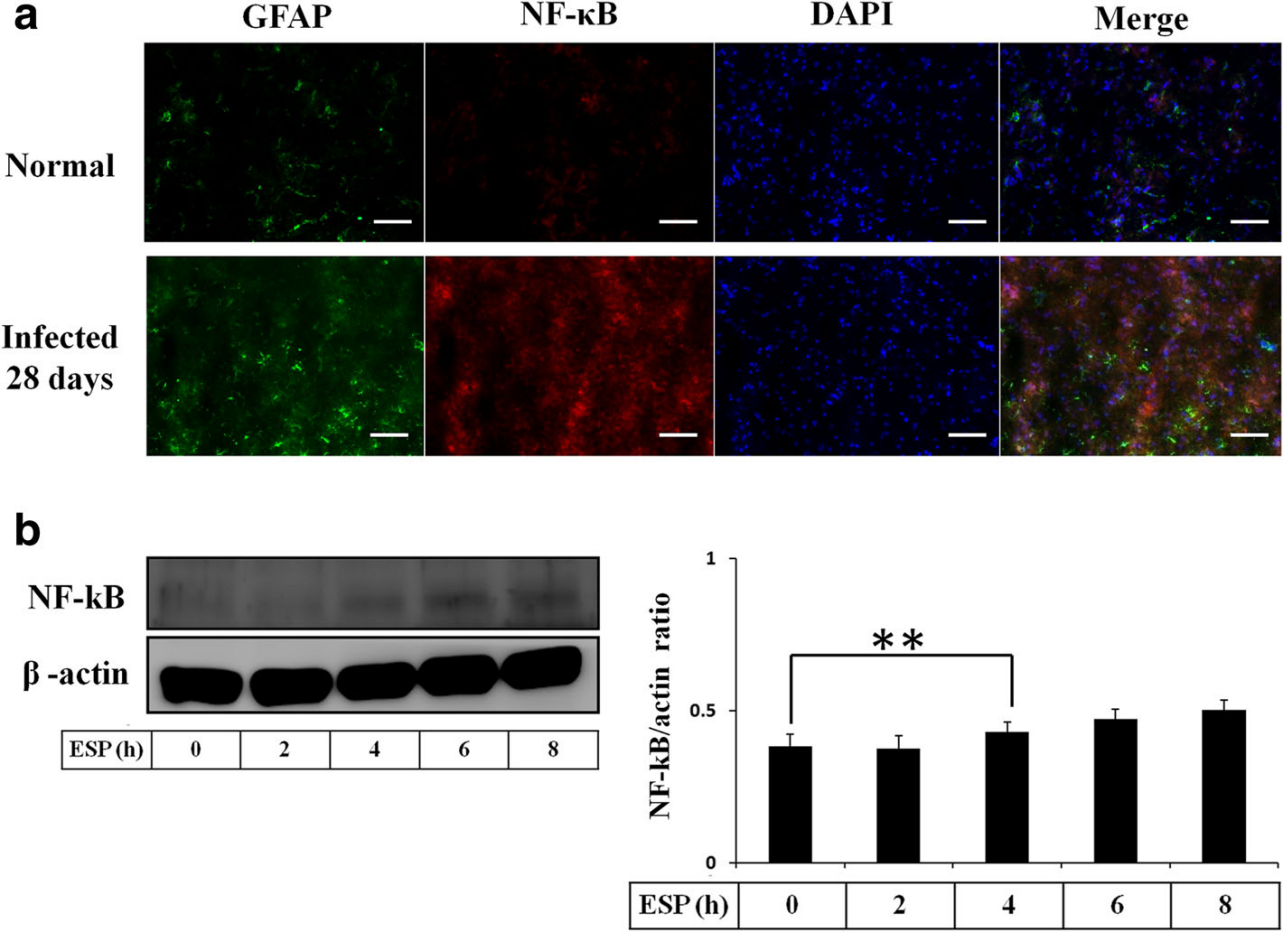
ESP induce NF-κB expression. **a** Fluorescence microscopy demonstrated the expression of NF-κB in astrocytes from the hippocampus of BALB/c mice infected with 25 *A. cantonensis* third-stage larvae on day 28 post-infection (GFAP: green; NF-κB: red; colocalization of NF-κB and Shh: yellow). **b** Western blot analysis of NF-κB in astrocytes treated with ESP for 0–8 h. Data are expressed as the mean ± SD from three independent experiments (**P < 0.01). *Scale-bars*: 100 μm

In viral infection, cytokines are elevated through the NF-kB-mediated pathway [6]. Moreover, the chemokines CCL2/MCP-1 and CCL7/MCP-7 were activated through NF-κB-dependent pathways in rat astrocytes [36]. Therefore, in this study, we detected NF-κB expression in histological brain sections of *A. cantonensis*-infected mice. Immunofluorescence staining with antibodies against GFAP and NF-κB showed that NF-κB was significantly increased in activated astrocytes from *A. cantonensis*-infected mice after 21 days (Fig. 6a). NF-κB was also significantly expressed in astrocytes following *A. cantonensis* L5 ESP treatment in vitro (*t*_(4)_ = 5.786, *P* < 0.01) (Fig. 6b). These data demonstrated that *A. cantonensis* L5 activates NF-κB expression in stimulated astrocytes through secretion of ESP in mice.

### NF-κB induces the expressions of IL-1β and IL-6 in mouse astrocytes *via* the Shh signaling pathway following ESP treatment

**Fig. 7:**
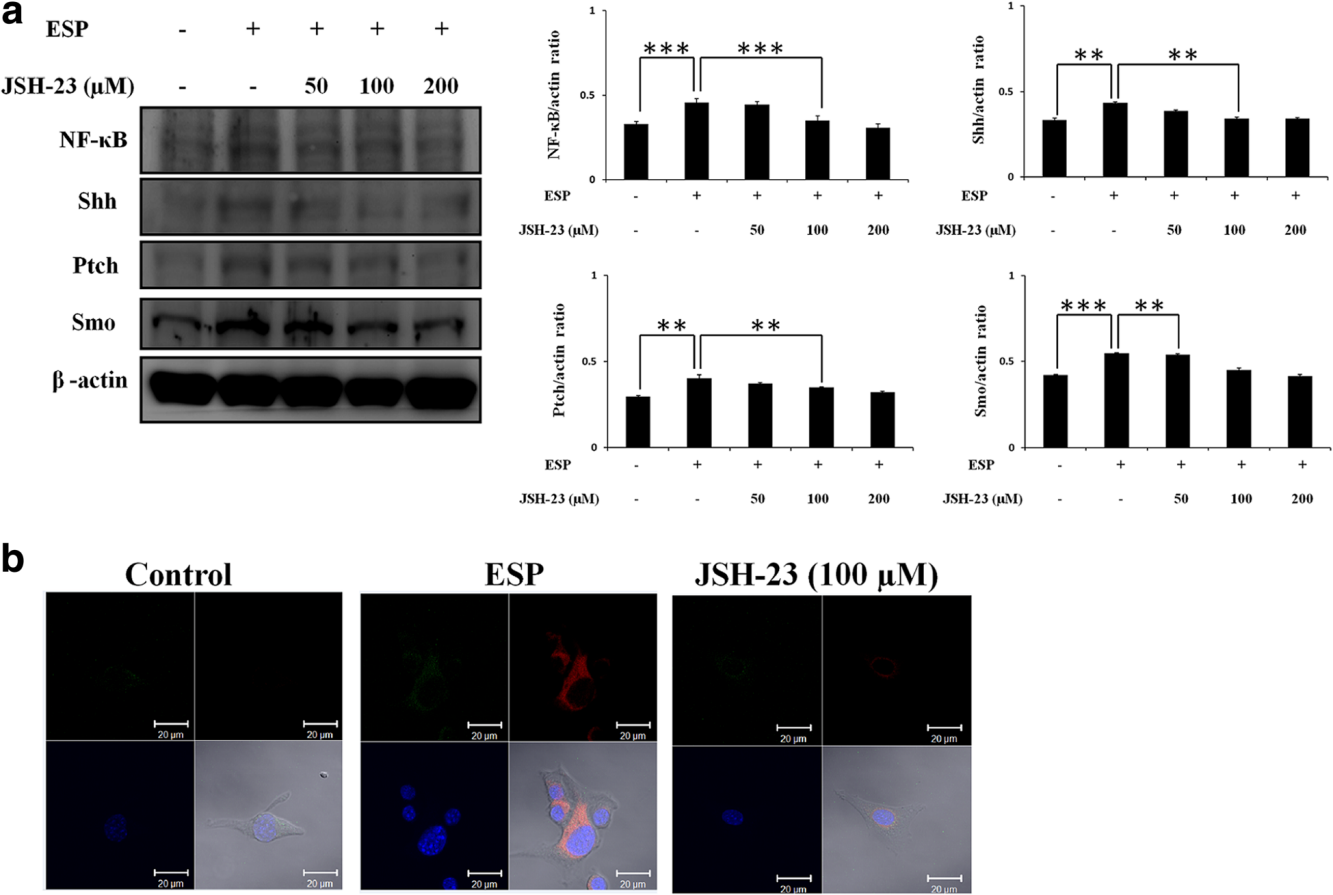
NF-κB induces Shh signaling pathway activation. **a** Western blot analysis of NF-κB, Shh, Ptch, and Smo in astrocytes co-cultured with ESP alone or JSH-23 for 2 h and then with 500 μg/ml ESP for 4 h; β-actin is shown as a control. Data are expressed as the mean ± SD from three independent experiments (**P < 0.01, ***P < 0.001). **b** The expression levels of Shh and GFAP in astrocytes. The Shh and GFAP protein expressions were determined by immunofluorescence staining of astrocytes with ESP alone or JSH-23 for 2 h and then with 500 μg/ml ESP for 4 h. (GFAP: green; Shh: red; DAPI: blue)

**Fig. 8:**
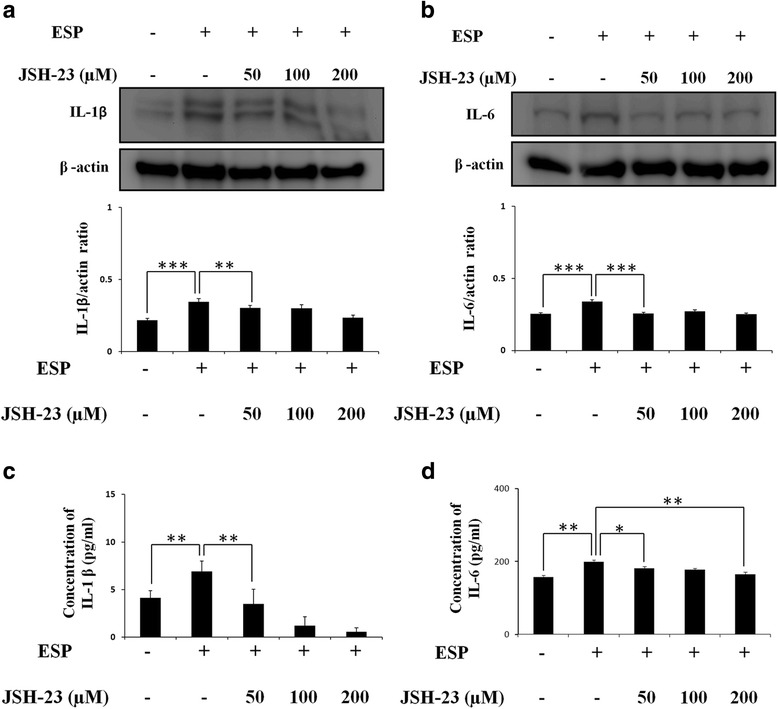
NF-κB induces the expressions of IL-1β and IL-6. Western blot analysis of IL-1β (**a**) and IL-6 (**b**) in astrocytes co-cultured with ESP alone or JSH-23 for 2 h and then with 500 μg/ml ESP for 4 h; β-actin is shown as a control. Data are expressed as the mean ± SD from three independent experiments (**P < 0.01, ***P < 0.001). **c**, **d** Changes in the concentrations of IL-1β and IL-6 protein in the culture medium of astrocytes detected by the ELISA. Data are expressed as the mean ± SD from three independent experiments (*P < 0.05, ** *P* < 0.01)

To investigate the effect of NF-κB on Shh signaling activation, we examined the expression of the Shh signaling-related molecules, including Shh, Ptch, Smo and Gli-1, in ESP-treated astrocytes with Western blotting. The Shh, Ptch, Smo and Gli-1 levels were significantly decreased in a concentration-dependent manner following pretreatment with the NF-κB inhibitor (JSH-23) (NF-κB: *t*_(4)_ = 9.855, *P* < 0.001; Shh: *t*_(4)_ = 6.192, *P* < 0.01; Ptch: *t*_(4)_ = 4.454, *P* < 0.01; Smo: *t*_(4)_ = 5.853, *P* < 0.01) (Fig. 7a). Immunofluorescence staining with antibodies against Shh and NF-κB showed that the expression of Shh has strongly decreased in NF-κB inhibitor-treated astrocytes (Fig. 7b). These data indicated that NF-κB activates the Shh signaling pathway in ESP treatment. Also, we also confirmed cytokine expression following inhibition of NF-κB. IL-1β and IL-6 generation (IL-1β: *t*_(4)_ = 5.133, *P* < 0.01; IL-6: *t*_(4)_ = 11.28, *P* < 0.001) (Fig. 8a, b) and secretion (IL-1β: *t*_(4)_ = 4.915, *P* < 0.01; IL-6: *t*_(4)_ = 7.22, *P* < 0.01) (Fig. 8c, d) were significantly decreased by inhibition of NF-κB in ESP-treated astrocytes. In conclusion, our results demonstrated cytokine expression in mouse astrocytes *via* the NF-κB/Shh signaling pathway following ESP treatment.

## Discussion

In *A. cantonensis* infection, reactive stress and injury are elevated in the parenchyma, meninges, and cerebrospinal fluid of the mouse brain [37, 38]. Moreover, this nematode induces blood-brain barrier dysfunction and immune responses in the host brain [39]. The ESP of *A. cantonensis* could induce eosinophil recruitment in the brain, but the function and effects of the ESP molecules are poorly understood [40]. The ESP are also important for studying the interaction between host and nematode, and these products are potential diagnostic markers for angiostrongyliasis [41]. However, the mechanisms of ESP-induced cytokine expression in astrocytes are largely unknown. In this study, we found that activated astrocytes from the nonpermissive host (mouse) secrete cytokines IL-1 and IL-6 following ESP treatment.

Astrocytes are important to brain cells that regulate immunocyte recruitment through the release of cytokines or chemokines, such as IL-1β and IL-6, in response to injuries or pathogen infections [42]. In *Toxoplasma gondii* infection, stimulated murine astrocytes can inhibit parasite infection and replication by the generation of cytokines [43, 44]. Moreover, some studies have shown that *T. gondii* tachyzoites or bradyzoites in astrocytes induce inflammatory cytokine (IL-1, IL-6, and TNF-α) generation in vitro [45]. IL-1β is a proinflammatory cytokine secreted by brain macrophages, astrocytes, and microglia, and it can also induce IL-6 production. IL-1β regulates blood-brain barrier function and permeability by induction of chemokines (CCL2, CCL20 or CXCL2) [8].

The Shh signaling pathway plays an important role in human development; it regulates the morphogenesis of tissues and organs [11]. In the CNS, the Shh pathway can stimulate CNS development, such as neural tube formation and brain cell proliferation [46–49]. This pathway is also activated in astrocytes by brain injury or H_2_O_2_ treatment, and Shh expression in astrocytes is higher than that in fibroblasts and neurons. Following H_2_O_2_ treatment, the astrocytes protect cortical neurons by secretion of Shh protein [2]. Moreover, our previous study showed that the Shh signaling pathway inhibits cell apoptosis in astrocytes by activation of GRP78 and Bcl-2 in *A. cantonensis* soluble antigen treatment [33]. In this study, we found that the expression of Shh signaling pathway proteins, such as SHH, PTCH, SMO, and GLI-1, were increased in astrocytes after *A. cantonensis* ESP treatment.

Neuroinflammation plays a major role in parasite infection, Parkinson’s disease and Alzheimer’s disease [50–53]. Several proinflammatory mediators in the brain could induce neuroinflammation, including cytokines and chemokines. Astrocytes and microglia are the major glial cells involved in activation of neuroimmunological responses in the CNS [54]. Several studies have shown that the expression of the transcription factor NF-κB is involved in astrocyte activation [55]. Furthermore, NF-κB has been shown to regulate the transcription of cytokines and chemokines [56]. NF-κB can induce astrocyte inflammation *via* the expressions of cytokines (IL-1β) and chemokines (MCP-1) [57]. In viral infection, cytokine expression is also elevated through a TNF-α/NF-κB-related pathway [6].

## Conclusions

Here, we investigated the molecular mechanisms of *A. cantonensis* ESP-stimulated expression of cytokines, including IL-1β and IL-6, in cultured mouse astrocytes. In conclusion, the present work showed that NF-κB induces the expressions of IL-1β and IL-6 through the Shh signaling pathway in ESP-treated astrocytes.

## Additional file

Additional file 1: Figure S1. The activation of Shh pathway in astrocytes co-cultured with cell medium. Astrocytes were treated with DMEM/F-12 at different time points. The protein levels of Shh, Ptch, Smo, and Gli-1 were determined in astrocytes by Western blots. Statistical significance was determined by Student’s *t* test (*n* = 3). (PDF 166 kb)

## Abbreviations

CNS: Central nervous system
Dhh: Desert hedgehog
DMEM/F-12: Dulbecco’s modified Eagle’s medium/F-12
ESP: Excretory/secretory products
FBS: Fetal bovine serum
Gli: Glioma-associated oncogene
Hh: Hedgehog
Ihh: Indian hedgehog
IL-1β: Interleukin-1β
L5: Fifth-stage larvae
PFA: Paraformaldehyde
Ptc: Patched
r-Shh: Recombinant Shh
SD: Sprague-Dawley
Shh: Sonic hedgehog
Smo: Smoothened
