# Supplementary material for: Stimulation of IL-1β and IL-6 through NF-κB and sonic hedgehog-dependent pathways in mouse astrocytes by excretory/secretory products of fifth-stage larval Angiostrongylus cantonensis
Source: Parasit Vectors. 2017 Sep 26;10:445. doi: 10.1186/s13071-017-2385-0 (PMC5615811; doi:10.1186/s13071-017-2385-0)
Supplement: Additional file 1: Figure S1. — The activation of Shh pathway in astrocytes co-cultured with cell medium. Astrocytes were treated with DMEM/F-12 at different time points. The protein levels of Shh, Ptch, Smo, and Gli-1 were determined in astrocytes by Western blots. Statistical significance was determined by Student’s t test (n = 3). (PDF 166 kb) [file 13071_2017_2385_MOESM1_ESM.pdf]

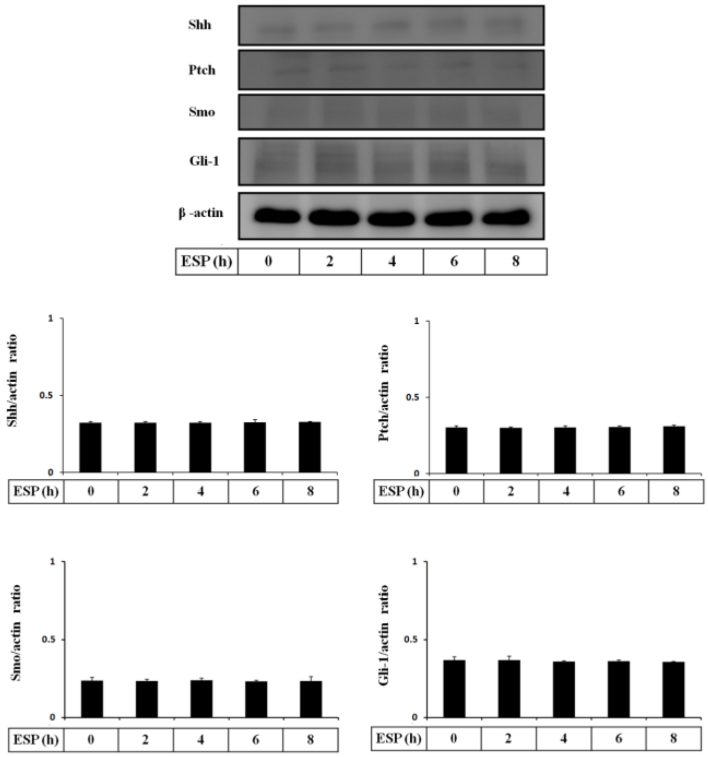

Figure S1. The activation of Shh pathway in astrocytes cocultured with cell medium.

Astrocytes were treated with DMEM/F-12 at different timepoints. The protein levels of Shh, Ptch, Smo and Gli-1 were determined in astrocytes by Western blots. Statistical significance was determined by Student’s t test. (n = 3).
